# Supplementary material for: The interactome of intact mitochondria by cross-linking mass spectrometry provides evidence for coexisting respiratory supercomplexes
Source: Mol Cell Proteomics. 2017 Dec 8;17(2):216–32. doi: 10.1074/mcp.RA117.000470 (PMC5795388; doi:10.1074/mcp.RA117.000470)
Supplement: Supplemental Data [file supp_RA117.000470_133922_0_supp_23978_fzffwf.zip › spectra_annotation/mito_DR_spectra_annotation/278-1-2-1-5-1.pdf]

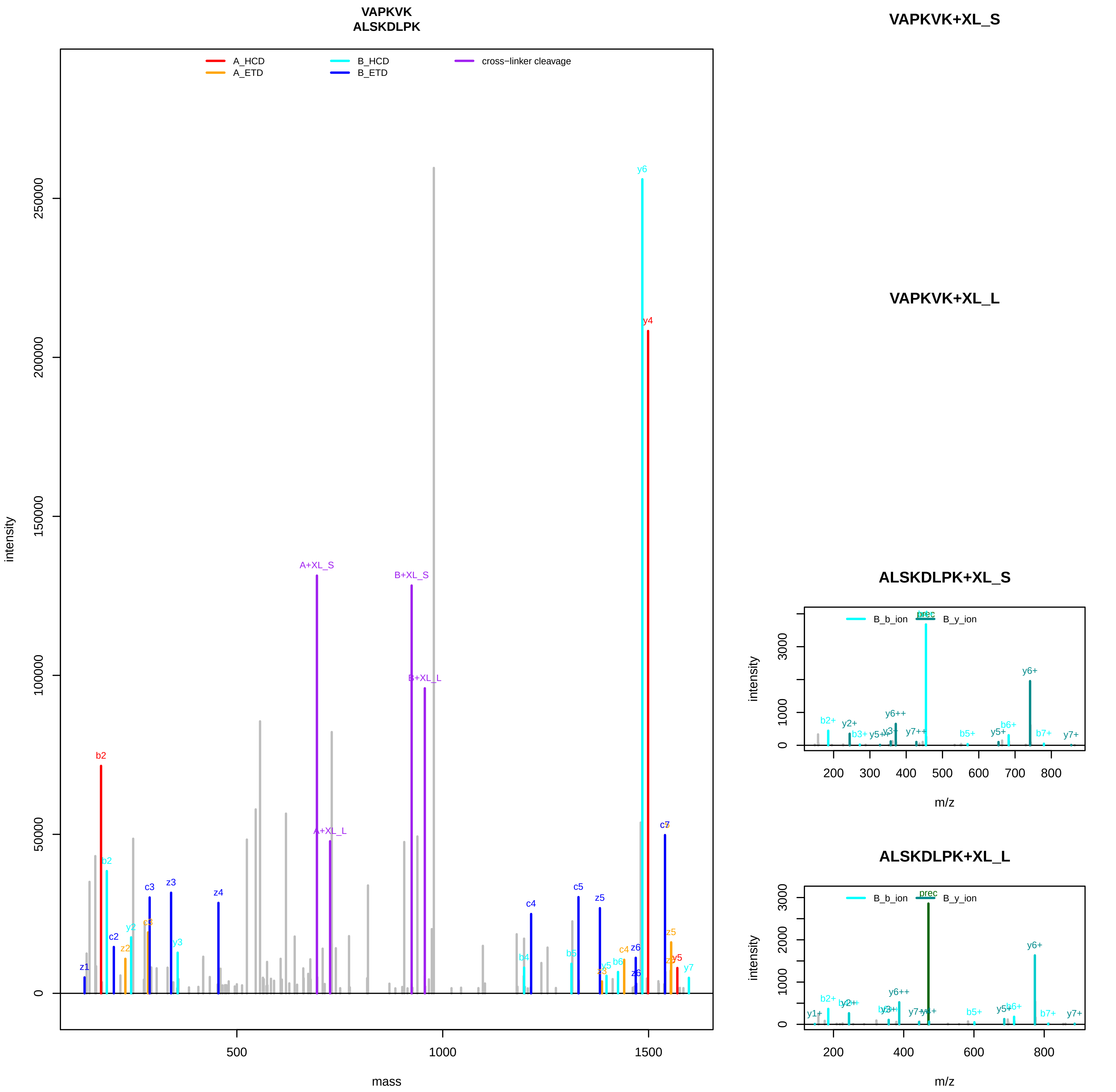
VAPKVK
ALSKDLPK
A_HCD
A_ETD
B_HCD
B_ETD
cross−linker cleavage
intensity
mass
y6
y4
A+XL_S
B+XL_S
B+XL_L
A+XL_L
b2
c7
z1
c2
y2
z2
c3
z3
y3
z4
c4
b4
c5
b5
z5
y5
b6
c4
z6
z3
z5
y5
y7
0
50000
100000
150000
200000
250000
500
1000
1500
VAPKVK+XL_S
VAPKVK+XL_L
ALSKDLPK+XL_S
B_b_ion
B_y_ion
prec
y6+
y6++
b2+
y2+
b3+
y5++
y3+
y7++
b5+
y5+
b6+
b7+
y7+
intensity
m/z
0
1000
3000
200
300
400
500
600
700
800
ALSKDLPK+XL_L
B_b_ion
B_y_ion
prec
y6+
y6++
b2+
y2+
b3+
y1+
y7+
b5+
y5+
b6+
b7+
y7+
intensity
m/z
0
1000
2000
3000
200
400
600
800
